# Antiproliferative Activity and Molecular Docking of Novel Double-Modified Colchicine Derivatives

**DOI:** 10.3390/cells7110192

**Published:** 2018-11-01

**Authors:** Urszula Majcher, Greta Klejborowska, Mahshad Moshari, Ewa Maj, Joanna Wietrzyk, Franz Bartl, Jack A. Tuszynski, Adam Huczyński

**Affiliations:** 1 Department of Bioorganic Chemistry, Faculty of Chemistry, Adam Mickiewicz University, Umultowska 89b, 61-614 Poznan, Poland; urszula.majcher@amu.edu.pl (U.M.); greta.klejborowska@amu.edu.pl (G.K.); 2 Department of Chemistry, University of Alberta, Edmonton, AB T6G 1Z2, Canada; moshari@ualberta.ca; 3 Hirszfeld Institute of Immunology and Experimental Therapy, Polish Academy of Sciences, Rudolfa Weigla 12, 53-114 Wrocław, Poland; ewa.maj@iitd.pan.wroc.pl (E.M.); wietrzyk@iitd.pan.wroc.pl (J.W.); 4 Institut für Biologie, AG Biophysikalische Chemie, Humboldt Universität zu Berlin, Invalidenstr, 42, 10099 Berlin, Germany; bartlfra@cms.hu-berlin.de; 5 Department of Oncology, University of Alberta, Edmonton, AB T6G 1Z2, Canada; jack.tuszynski@gmail.com

**Keywords:** colchicine binding site inhibitor, β-tubulin affinity, antimitotic agent, antiproliferative activity, thiocolchicine

## Abstract

Microtubules are tubulin polymer structures, which are indispensable for cell growth and division. Its constituent protein β-tubulin has been a common drug target for various diseases including cancer. Colchicine has been used to treat gout, but it has also been an investigational anticancer agent with a known antimitotic effect on cells. However, the use of colchicine as well as many of its derivatives in long-term treatment is hampered by their high toxicity. To create more potent anticancer agents, three novel double-modified colchicine derivatives have been obtained by structural modifications in C-4 and C-10 positions. The binding affinities of these derivatives of colchicine with respect to eight different isotypes of human β-tubulin have been calculated using docking methods. In vitro cytotoxicity has been evaluated against four human tumor cell lines (A549, MCF-7, LoVo and LoVo/DX). Computer simulations predicted the binding modes of these compounds and hence the key residues involved in the interactions between tubulin and the colchicine derivatives. Two of the obtained derivatives, 4-bromothiocolchicine and 4-iodothiocolchicine, were shown to be active against three of the investigated cancer cell lines (A549, MCF-7, LoVo) with potency at nanomolar concentrations and a higher relative affinity to tumor cells over normal cells.

## 1. Introduction

Microtubules, present in all eukaryotic cells, are cylindrical polymers composed of α/β-tubulin heterodimers. They are involved in a wide range of key cellular processes, such as the maintenance of cellular morphology and the active motor transport of cellular components throughout the cytoplasm [1]. Another essential role microtubules play is the formation of mitotic spindles and force generation during mitosis with the purpose of separating chromosomes [2]. A failure within this mitotic spindle apparatus leads to mitotic arrest and eventually apoptosis. This results in cell death, which is a desirable outcome for cancer cells, but not for healthy tissues. With the objective of promoting the former and avoiding the latter effect, microtubules have become the target for a large number of antimitotic agents that act by either favoring or inhibiting microtubule polymerization by binding at specific sites on the exposed surface of α/β-tubulin heterodimers [3,4,5,6,7]. Although there are multiple distinct binding sites on a tubulin heterodimer, β-tubulin is the main binding partner for all major microtubule-targeting drug families [8,9,10].

Among them colchicine (**1**), a well-known tropolone alkaloid isolated from *Colchicum autumnale*, is of particular interest due to its powerful antimitotic properties. It has played an important role in studies of mitosis and the therapeutic potential of using the colchicine binding site on β-tubulin in chemotherapy applications has generated much interest [5,6,7,11,12,13,14,15,16]. However, colchicine itself as well as many of its derivatives, have not yet been used as successful drugs in long-term treatment because of their detrimental side effects [6,7,11]. Up to now, many structure-activity relationship studies have been performed to elucidate the structural features required for tubulin binding. These studies have demonstrated great importance of the 9-keto function and the methoxy groups at C-1, C-2, and C-10 as well as the importance of stereochemistry of 7-acetamido center, which is critical for antimitotic activity. Ring B appears to be responsible for the irreversible nature of colchicine binding to tubulin, although it may also contribute to its toxic effects [11,17]. Therefore, currently much interest has focused on structural modification of **1** in the hope of improving its anticancer activity [18,19,20,21,22,23,24,25,26,27,28,29,30,31,32,33].

In 2011 Yasobu et al. published results of their studies on C-4 halogen substituted colchicine derivatives [32]. On the evaluation of cell-growth inhibitory activity using mice transplanted with the HCT116 human colorectal carcinoma cell line, some of the derivatives exhibited less toxicity in mice and more potent cell-growth inhibitory activity than **1**. Moreover, another colchicine derivative with thiomethyl group at C-10 called thiocolchicine, is also a potent inhibitor of tubulin polymerization and cell growth, and binds to tubulin more rapidly than colchicine [34,35,36]. Thiocolchicine is not only easily available from colchicine after treatment with sodium methanethiolate, but also is more stable, which allows for using harsher reaction conditions without formation of isomers.

Inspired by these reports, we decided to verify how double modification in C-4 and C-10 positions influences the activity and selectivity of colchicine. Below, we report the synthesis and spectroscopic analysis of a series of seven compounds, of which three are entirely novel compounds synthesized for the first time. We also provide an evaluation of these derivatives as cytotoxic, tubulin-targeting agents. The antiproliferative effect of seven colchicines derivatives (**2**–**8**) was tested in vitro using four cancer cell lines and one normal murine embryonic fibroblast cell line. To better understand the interactions between these colchicine derivatives and various isotypes of β-tubulin, we investigated potential binding modes of novel double-modified derivatives, 4-halocolchicines as well as colchicine docked into the colchicine binding site (CBS) of eight different isotypes of β-tubulin using AutoDock4 software (version 2018.2.0, Tableau Research, Standford University, Seattle, WA, USA) under flexible ligand and rigid receptor condition. A detailed discussion regarding differences between the structures of the synthesized compounds and their ability to form complexes with CBS is provided below.

## 2. Materials and Methods

### 2.1. General

All precursors for the synthesis and solvents were obtained from Sigma-Aldrich (Merck KGaA, Saint Louis, MO, USA) and were used as received without further purification. CDCl_3_ spectral grade solvent was stored over 3 Å molecular sieves for several days. Thin layer chromatographywas carried out on precoated plates (TLC silica gel 60 F254, Aluminum Plates Merck (Merck KGaA Saint Louis, MO, USA)) and spots were detected by illumination with an ultra-violet (UV) lamp. All the solvents used in flash chromatography were of HPLC grade (CHROMASOLV from Sigma-Aldrich, Merck KGaA, Saint Louis, MO, USA) and were used as received. The elemental analysis of compounds was carried out on Vario ELIII (Elementar, Langenselbold, Germany).

### 2.2. Spectroscopic Measurements

The ^1^H, ^13^C spectra were recorded on a Varian VNMR-S 400 MHz spectrometer (Varian, Inc., Palo Alto, CA, USA). ^1^H-NMR measurements of **2**–**8** (0.07 mol dm^−3^) in CDCl_3_ were carried out at the operating frequency 402.64 MHz. The error of the chemical shift value was 0.01 ppm. The ^13^C-NMR spectra were recorded at the operating frequency 101.25 MHz. The error of chemical shift value was 0.1 ppm. All spectra were locked to deuterium resonance of CDCl_3_. The ^1^H and ^13^C-NMR spectra are shown in the Supplementary Materials.

The FT-IR spectra of **2**–**8** in the mid infrared region were recorded in KBr. The spectra were taken with an IFS 113v FT-IR spectrophotometer (Bruker, Karlsruhe, Germany) equipped with a deuterated triglycine sulfate detector (DTGS) detector; resolution 2 cm^–1^, NSS = 64. The Happ-Genzel apodization function was used.

The ESI (Electrospray Ionization) mass spectra were recorded also on a Waters/Micromass (Waters Corporation, Manchester, UK) ZQ mass spectrometer equipped with a Harvard Apparatus syringe pump. The samples were prepared in dry acetonitrile (5 × 10^−5^ mol dm^−3^). The sample was infused into the ESI source using a Harvard pump at a flow rate of 20 mL min^−1^. The ESI source potentials were: capillary 3 kV, lens 0.5 kV, extractor 4 V. The standard ESI mass spectra were recorded at the cone voltages: 10 and 30 V. The source temperature was 120 °C and the desolvation temperature was 300 °C. Nitrogen was used as the nebulizing and desolvation gas at flow-rates of 100 dm^3^ h^−1^. Mass spectra were acquired in the positive ion detection mode with unit mass resolution at a step of 1 *m*/*z* unit. The mass range for ESI experiments was from *m*/*z* = 100 to *m*/*z* = 1000, as well as from *m*/*z* = 200 to *m*/*z* = 1500.

### 2.3. Synthesis

#### 2.3.1. Synthesis of **2**

To a mixture of **1** (500 mg, 1.25 mmol) in MeOH/water (1/1, *v*/*v*, 5 mL), the sodium methanethiolate (solution 21% in H_2_O, 0.83 mL, 2.5 mmol) was added. The mixture was stirred in at RT for 72 h. Reaction time was determined by TLC. After that time, the reaction mixture was quenched by the addition of water (150 mL). The whole mixture was extracted four times with CH_2_Cl_2_, and the combined organic layers were dried over MgSO_4_, filtered, and evaporated under reduced pressure. The residue was purified by CombiFlash^®^ (hexane/EtOAc (1/1), then EtOAc/MeOH, increasing concentration gradient) to give 2 with yield 78% [34].

The synthesis of compounds **4**, **6** and **8** was carried out analogously to the above starting respectively from the compounds **3**, **5** and **7**.

*Compound***2**, ^1^H-NMR (403 MHz, CDCl_3_) δ 7.92 (s, 1H), 7.46 (s, 1H), 7.33 (d, *J* = 10.4 Hz, 1H), 7.10 (d, *J* = 10.5 Hz, 1H), 6.55 (s, 1H), 4.72–4.64 (m, 1H), 3.95 (s, 3H), 3.91 (s, 3H), 3.67 (s, 3H), 2.54 (dd, *J* = 13.0, 5.8 Hz, 1H), 2.45 (s, *J* = 5.7 Hz, 3H), 2.43–2.26 (m, 2H), 1.99 (s, 3H), 1.94 (dd, *J* = 11.8, 5.5 Hz, 1H) ppm; ^13^C-NMR (101 MHz, CDCl_3_) δ 182.4, 170.0, 158.1, 153.6, 151.8, 151.1, 141.6, 138.6, 134.8, 134.4, 128.3, 126.7, 125.6, 107.3, 61.6, 61.4, 56.1, 52.3, 36.4, 29.9, 22.8, 15.1 ppm. FT-IR (KBr pellet): 3283, 2935, 1660, 1605, 1541, 1485, 1461, 1425, 1404, 1349, 1321, 1286, 1236, 1195, 1155, 1138, 1095, 1023 cm^−1^. ESI-MS (*m*/*z*): [M + H]^+^ calcd. 416, found 416, [M + Na]^+^ calcd. 438, found 438, [M + K]^+^ calcd. 454 found 454, [2M + Na]^+^ calcd. 853, found 853, [3M + Na]^+^ calcd. 1268, found 1268.

*Compound***4**, Amorphous yellow solid. ^1^H-NMR (403 MHz, CDCl_3_) δ 7.98 (d, *J* = 6,7 Hz, 1H), 7.44 (s, 1H), 7.26 (d, *J* = 10.3 Hz, 1H), 7.08 (d, *J* = 10.8 Hz, 1H), 4.58 (dt, *J* = 13.1, 6.7 Hz, 1H), 3.98 (s, 3H), 3.96 (s, 3H), 3.61 (s, 3H), 3.24 (dd, *J* = 13.5, 4.8 Hz, 1H), 2.44 (s, 3H), 2.27 (ddd, *J* = 18.0, 12.1, 6.0 Hz, 1H), 2.14 (td, *J* = 13.4, 6.2 Hz, 1H), 2.00 (s, 3H), 1.92–1.80 (m, 1H); ^13^C-NMR (101 MHz, CDCl_3_) δ 182.4, 170.1, 159.1, 151.3, 150.2, 149.7, 146.6, 137.3, 134.8, 131.7, 129.9, 128.1, 126.4, 122.1, 61.6, 61.5, 61.1, 52.2, 34.5, 25.9, 22.8, 15.1 ppm. FT-IR (KBr pellet): 3290, 2936, 1661, 1608, 1550, 1464, 1413, 1349, 1327, 1288, 1267, 1197, 1140, 1086, 1023 cm^−1^. ESI-MS (*m*/*z*): [M + H]^+^ calcd. 450, found 450, [M + Na]^+^ calcd. 472, found 472, [2M + H]^+^ calcd. 889, found 889, [2M + Na]^+^ calcd. 921, found 921. Anal. Calcd. for C, 58.73; H, 5.38; Cl, 7.88; N, 3.11; O, 17.78; S, 7.13; found: C, 58.61; H 5.35; Cl, 7.93; N, 3.01; S, 7.25.

*Compound***6**, Amorphous yellow solid. ^1^H-NMR (403 MHz, CDCl_3_) δ 7.68 (d, *J* = 6.6 Hz, 1H), 7.42 (s, 1H), 7.26 (d, *J* = 9.6 Hz, 1H), 7.08 (d, *J* = 10.8 Hz, 1H), 4.61–4.52 (m, 1H), 3.99 (s, 3H), 3.97 (s, 3H), 3.63 (s, 3H), 3.27 (d, *J* = 8.0 Hz, 1H), 2.45 (s, 3H), 2.25 (dt, *J* = 13.4, 7.9 Hz, 2H), 2.01 (s, *J* = 1.6 Hz, 3H), 1.85 (dd, *J* = 6.7, 4.1 Hz, 1H) ppm; ^13^C-NMR (101 MHz, CDCl_3_) δ 182.4, 170.0, 159.2, 151.2, 151.0, 150.4, 146.6, 137.4, 134.8, 133.4, 130.2, 128.1, 126.3, 113.5, 61.6, 61.5, 61.0, 52.2, 34.5, 29.0, 22.9, 15.2 ppm. FT-IR (KBr pellet): 3267, 2930, 1659, 1603, 1559, 1462, 1410, 1347, 1138, 1074, 1053, 1014 cm^−1^. ESI-MS (*m*/*z*): [M + H]^+^ calcd. 494, found 494, [M + 2 + H]^+^ 496, found 496, [M + Na]^+^ calcd. 516, found 516, [M + 2 + Na]^+^ calcd. 518, found 518, [2M + H]^+^ calcd. 989, found 989, [2M + 2 + H]^+^ calcd. 991, found 991, [2M + Na]^+^ calcd. 1011, found 1011, [2M + 2 + Na]^+^ calcd. 1013, found 1013. Anal. Calcd. for C, 53.45; H, 4.89; Br, 16.16; N, 2.83; O, 16.18; S, 6.49; found: C, 53.56; H 4.81; Br, 16.28; N, 2.89; S, 6.55.

*Compound***8**, Amorphous yellow solid. ^1^H-NMR (403 MHz, CDCl_3_) δ 7.75 (d, *J* = 6.9 Hz, 1H), 7.42 (s, 1H), 7.25 (d, *J* = 10.3 Hz, 1H), 7.09 (d, *J* = 10.8 Hz, 1H), 4.58–4.50 (m, 1H), 3.97 (s, 3H), 3.95 (s, 3H), 3.63 (s, 3H), 3.18 (dd, *J* = 13.7, 5.0 Hz, 1H), 2.46 (s, 3H), 2.40 (dd, *J* = 13.6, 6.2 Hz, 1H), 2.32–2.23 (m, 1H), 2.01 (s, 3H), 1.85–1.79 (m, 1H); ^13^C-NMR (101 MHz, CDCl_3_) δ 182.4, 170.1, 159.1, 153.5, 151.4, 151.1, 145.6, 137.8, 136.8, 134.7, 129.7, 128.1, 126.3, 92.2, 61.6, 61.4, 60.8, 52.1, 34.5, 34.4, 22.9, 15.2 ppm. FT-IR (KBr pellet): 3288, 2936, 1660, 1607, 1547, 1461, 1406, 1346, 1318, 1288, 1262, 1197, 1138, 1081, 1019 cm^−1^. ESI-MS (*m*/*z*): [M + H]^+^ calcd. 542, found 542, [M + Na]^+^ calcd. 564, found 564, [M + K]^+^ calcd. 580, found 580. Anal. Calcd. for C, 48.81; H, 4.47; I, 23.44; N, 2.59; O, 14.78; S, 5.92; found: C, 48.67; H 4.55; I, 23.59; N, 2.64; S, 5.98.

#### 2.3.2. Synthesis of **3**

A mixture of *N*-chlorosuccinimide (175 mg, 1.31 mmol) and **1** (500 mg, 1.25 mmol) in acetonitrile was stirred at RT under nitrogen atmosphere for the 72 h. Reaction time was determined by TLC. The reaction was quenched with saturated aqueous Na_2_S_2_O_3_. The whole mixture was extracted four times with CH_2_Cl_2_, and the combined organic layers were dried over MgSO_4_, filtered, and evaporated under reduced pressure. The residue was purified by CombiFlash^®^ (EtOAc/MeOH, increasing concentration gradient) to give **3** with yield 75% [32].

^1^H-NMR (403 MHz, CDCl_3_) δ 8.29 (d, *J* = 6.2 Hz, 1H), 7.59 (s, 1H), 7.30 (d, *J* = 10.7 Hz, 1H), 6.87 (d, *J* = 11.2 Hz, 1H), 4.60–4.49 (m, 1H), 4.01 (s, 3H), 3.97 (s, 3H), 3.95 (s, 3H), 3.61 (s, 3H), 3.23 (dd, *J* = 13.7, 5.1 Hz, 1H), 2.31 (dq, *J* = 18.7, 6.2 Hz, 1H), 2.18–2.09 (m, 1H), 1.96 (s, 3H), 1.93–1.82 (m, 1H) ppm; ^13^C-NMR (101 MHz, CDCl_3_) δ 179.5, 170.2, 164.3, 152.0, 150.1, 149.7, 146.6, 135.8, 135.8, 131.7, 130.1, 129.8, 122.1, 112.5, 61.5, 61.5, 61.1, 56.5, 52.7, 34.5, 25.8, 22.7 ppm. FT-IR (KBr pellet): 3256, 2935, 1663, 1618, 1591, 1556, 1456, 1412, 1397, 1351, 1290, 1272, 1243, 1171, 1136, 1080, 1021 cm^−1^. ESI-MS (*m*/*z*): [M + H]^+^ calcd. 434, found 434, [M + Na]^+^ calcd. 456, found 456, [2M + Na]^+^ calcd. 889, found 889.

#### 2.3.3. Synthesis of **5**

A mixture of *N*-bromosuccinimide (279 mg, 1.57 mmol) and **1** (500 mg, 1.25 mmol) in acetonitrile was stirred at RT under nitrogen atmosphere for the 72 h. Reaction time was determined by TLC. The reaction was quenched with saturated aqueous Na_2_S_2_O_3_. The whole mixture was extracted four times with CH_2_Cl_2_, and the combined organic layers were dried over MgSO_4_, filtered, and evaporated under reduced pressure. The residue was purified by CombiFlash^®^ (EtOAc/MeOH, increasing concentration gradient) to give **5** with yield 95% [32].

^1^H-NMR (403 MHz, CDCl_3_) δ 8.02 (s, 1H), 7.58 (s, 1H), 7.30 (d, *J* = 10.7 Hz, 1H), 6.88 (d, *J* = 11.1 Hz, 1H), 4.59–4.49 (m, 1H), 4.03 (s, 3H), 3.99 (s, 3H), 3.96 (s, 3H), 3.63 (s, 3H), 3.27 (dd, *J* = 13.0, 4.3 Hz, 1H), 2.26 (dd, *J* = 13.1, 5.2 Hz, 1H), 2.18 (d, *J* = 2.4 Hz, 1H), 1.99 (s, 3H), 1.78 (s, 1H) ppm; ^13^C-NMR (101 MHz, CDCl_3_) δ 179.5, 170.2, 164.4, 151.8, 151.1, 150.4, 146.6, 135.8, 135.7, 133.4, 130.2, 130.0, 113.5, 112.4, 61.5, 61.5, 61.0, 56.5, 52.6, 34.5, 28.9, 22.8 ppm. FT-IR (KBr pellet): 3274, 2936, 1662, 1617, 1589, 1565, 1462, 1411, 1398, 1350, 1270, 1250, 1172, 1137, 1080, 1018 cm^−1^. ESI-MS (*m*/*z*): [M + Na]^+^ calcd. 500, found 500, [M + 2 + Na]^+^ calcd. 502, found 502, [2M + 2 + Na]^+^ calcd. 979, found 979, [2M + Na]^+^ calcd. 977, found 977, [2M + 4 + Na]^+^ calcd. 981, found 981.

#### 2.3.4. Synthesis of **7**

A mixture of *N*-iodosuccinimide (560 mg, 2.49 mmol) and **1** (500 mg, 1.25 mmol) in AcOH was stirred at 70 °C under nitrogen atmosphere for the 20 h. Reaction time was determined by TLC. The reaction was quenched with saturated aqueous Na_2_S_2_O_3_. The whole mixture was extracted four times with CH_2_Cl_2_, and the combined organic layers were dried over MgSO_4_, filtered, and evaporated under reduced pressure. The residue was purified by CombiFlash^®^ (EtOAc/MeOH, increasing concentration gradient) to give **7** with yield 95% [32].

^1^H-NMR (403 MHz, CDCl_3_) δ 8.22 (d, *J* = 5.6 Hz, 1H), 7.61 (s, 1H), 7.30 (d, *J* = 10.7 Hz, 1H), 6.89 (d, *J* = 11.2 Hz, 1H), 4.55–4.47 (m, 1H), 4.04 (s, 3H), 3.97 (s, 3H), 3.95 (s, 3H), 3.63 (s, 3H), 3.21–3.15 (m, 1H), 2.40 (dd, *J* = 12.7, 5.0 Hz, 1H), 1.99 (s, 3H), 1.87–1.81 (m, 1H); ^13^C-NMR (101 MHz, CDCl_3_) δ 179.5, 170.2, 164.4, 153.4, 152.0, 151.4, 145.6, 136.7, 136.2, 135.6, 130.1, 129.5, 112.5, 92.1, 61.5, 61.3, 60.7, 56.5, 52.6, 34.4, 34.4, 22.7 ppm; FT-IR (KBr pellet): 3274, 2934, 1662, 1617, 1588, 1563, 1461, 1406, 1393, 1346, 1318, 1266, 1249, 1171, 1136, 1078, 1015 cm^−1^. ESI-MS (*m*/*z*): [M + H]^+^ calcd. 526, found 526 [M + Na]^+^ calcd. 548, found 548.

### 2.4. Antiproliferative Activity of Colchicine and Its Derivatives

Four human cancer cell lines and one murine normal cell line were used to evaluate antiproliferative activity of colchicine and its derivatives: human lung adenocarcinoma (A549), human breast adenocarcinoma (MCF-7), human colon adenocarcinoma cell lines sensitive and resistant to doxorubicin (LoVo) and (LoVo/DX) respectively, and normal murine embryonic fibroblast cell line (BALB/3T3). The BALB/3T3 cell line was purchased from the American Type Culture Collection (ATCC, Manassas, VA, USA), A549 and MCF-7 cell lines—from European Collection of Authenticated Cell Cultures (Salisbury, UK), LoVo cell line was purchased from the ATCC (ATCC, Manassas, VA, USA), and LoVo/DX by courtesy of Prof. E. Borowski (Technical University of Gdańsk, Gdańsk, Poland). All the cell lines are maintained in the Institute of Immunology and Experimental Therapy (IIET), Wroclaw, Poland. Human lung adenocarcinoma cell line was cultured in mixture of OptiMEM and RPMI 1640 (1:1) medium (IIET, Wroclaw, Poland), supplemented with 5% fetal bovine serum (GE Healthcare, Logan, UT, USA) and 2 mM l-glutamine (Sigma-Aldrich, Merck KGaA, Saint Louis, MO, USA). Human breast adenocarcinoma cell line was cultured in mixture of Eagle medium (IIET, Wroclaw, Poland), supplemented with 10% fetal bovine serum, 2 mM l-glutamine, 8 μg/mL insulin and 1% amino-acids (Sigma-Aldrich, Merck KGaA, Saint Louis, MO, USA). Human colon adenocarcinoma cell lines were cultured in mixture of OptiMEM and RPMI 1640 (1:1) medium (IIET, Wroclaw, Poland), supplemented with 5% fetal bovine serum (GE Healthcare, Logan UT, USA), 2 mM l-glutamine, 1 mM sodium pyruvate (Sigma-Aldrich, Merck KGaA, Saint Louis, MO, USA) and 10 μg/100 mL doxorubicin for LoVo/DX (Sigma-Aldrich, Merck KGaA, Saint Louis, MO, USA). Murine embryonic fibroblast cells were cultured in Dulbecco medium (Life Technologies Limited, Paisley, UK), supplemented with 10% fetal bovine serum (GE Healthcare, Logan, UT, USA) and 2 mM glutamine (Sigma-Aldrich, Merck KGaA, Saint Louis, MO, USA). All culture media contained antibiotics: 100 U/mL penicillin and 100 μg/mL streptomycin (Polfa-Tarchomin, Warsaw, Poland). All cell lines were cultured during entire experiment in humid atmosphere at 37 °C and 5% CO_2_. Cells were tested for mycoplasma contamination by mycoplasma detection kit for conventional PCR: Venor GeM Classic (Minerva Biolabs GmbH, Berlin, Germany) and negative results was obtained. The procedure is repeated every year or in the case of less frequently used lines: after thawing.

#### 2.4.1. The Antiproliferative Assays In Vitro

Twenty-four hours before adding the tested compounds, all cell lines were seeded in 96-well plates (Sarstedt, Nümbrecht, Germany) in appropriate media with 10^4^ cells per well. All cell lines were exposed to each tested agent at four different concentrations in the range 100–0.01 μg/mL for 72 h. Cells were also exposed to the reference drug cisplatin (Teva Pharmaceuticals Polska, Warsaw, Poland) and doxorubicin (Accord Healthcare Limited, Middlesex, UK). Additionally, all cell lines were exposed to DMSO (solvent used for tested compounds) (POCh, Gliwice, Poland) at concentrations corresponding to those present in tested agents’ dilutions. After 72 h sulforhodamine B assay (SRB) was performed [37].

#### 2.4.2. SRB

After 72 h of incubation with the tested compounds, cells were fixed in situ by gently adding of 50 μL per well of cold 50% trichloroacetic acid TCA (POCh, Gliwice, Poland) and were incubated at 4 °C for one hour. Following, wells were washed four times with water and air dried. Next, 50 μL of 0.1% solution of sulforhodamine B (Sigma-Aldrich, Merck KGaA, Saint Louis, MO, USA) in 1% acetic acid (POCh, Gliwice, Poland) were added to each well and plates were incubated at room temperature for 0.5 h. After incubation time, unbound dye was removed by washing plates four times with 1% acetic acid whereas stain bound to cells was solubilized with 10 mM Tris base (Sigma-Aldrich, Steinheim, Germany). Absorbance of each solution was read at Synergy H4 Hybrid Multi-Mode Microplate Reader (BioTek Instruments, Inc., Winooski, VT, USA) at the 540 nm wavelength.

Results are presented as mean IC_50_ (concentration of the tested compound, that inhibits cell proliferation by 50%) ± standard deviation. IC_50_ values were calculated in Cheburator 0.4, Dmitry Nevozhay software (version 1.2.0 software by Dmitry Nevozhay, 2004–2014, http://www.cheburator.nevozhay.com, freely available) for each experiment [38]. Compounds at each concentration were tested in triplicates in single experiment and each experiment was repeated at least three times independently. Results are summarized in Table 1. The Resistance Index (*RI*) was defined as the ratio of IC_50_ for a given compound calculated for resistant cell line to that measured for its parental drug sensitive cell line (Table 1).

### 2.5. Molecular Docking Simulations

A combination of different theoretical methods was used to explore ligand-tubulin interactions. The ligand structures were first minimized and then fully optimized based on the RHF/cc-pVDZ level of theory in GAMESS-US version 2010-10-01. Since there is no crystal structure available for human βI tubulin (TBB5_HUMAN), we obtained its sequence from UniProt (ID: Q13509). We used the tubulin structure 1SA0.pdb as a template to construct the homology model for βI tubulin using MOE2015. We then docked the small library of colchicine derivatives to the protein using the AutoDock4 program under flexible ligand and rigid receptor conditions (Table 2). AutoDock4 software (version 2018.2.0, Tableau Research, Standford University, Seattle, WA, USA) is designed to predict how drug candidates bind to a receptor of a known 3D structure and consists of two main programs: AutoDock performs the docking of the ligand to a set of grids describing the target protein; AutoGrid pre-calculates these grids. The estimated Moriguchi octanol-water partition coefficient, MlogP, of the compounds were calculated by ADMET Predictor 8.0 (ADMET Predictor, Simulations Plus, Lancaster, CA, USA).

## 3. Results

### 3.1. Chemistry

**Scheme 1:**
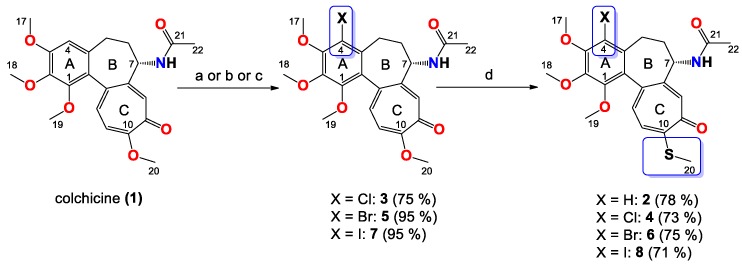
Synthesis of colchicine derivatives (**2**–**8**). Reagents and conditions: (a) for **3**, NCS, acetonitrile, RT; (b) for **5**, NBS, acetonitrile, RT; (**c**) for **7**, NIS, AcOH, 70 °C; (**d**) MeOH/H_2_O, CH_3_SNa, RT.

The synthetic routes to colchicines derivatives **2**–**8** are outlined in Scheme 1. Colchicine (**1**) was treated with sodium methanethiolate to give thiocolchicine (**2**) with yield 78% according to the previously described method [34]. 4-chlorocolchicine (**3**), 4-bromocolchicine (**5**), and 4-iodocolchicine (**7**) were synthesized from **1** by treatment with NCS, NBS, and NIS with yields from 75% up to 95%, respectively, based on the methods developed earlier [32]. For 4-chlorocolchicine (**3**) and 4-bromocolchicine (**5**), the application of milder conditions, i.e., the replacement of acetic acid (the solvent) by acetonitrile followed by reacting at room temperature, also allowed to obtain the same final yields. Compounds **3**, **5**, **7** were then treated with sodium methanethiolate to give double-modified derivatives (**4**, **6**, **8**) with yields from 71% to 75%. 

The structures of all products **2**–**8** were determined using the elemental analysis, ESI-MS, FT-IR, ^1^H- and ^13^C-NMR methods and are shown in Supplementary Material. In the ^13^C-NMR spectra of the 4-halo derivatives a resonance for the C-4 carbon atom of the A aromatic ring was observed at 122.1 ppm for **3**, at 113.5 ppm for **5** and at 92.1 ppm for **7**, while in **1** it was observed at 107.3 ppm. After the introduction of thiomethyl group in C-10 positions shifts of the signal for the C-20 carbon atom in compounds **2**, **4**, **6** and **8** were observed in the range 15.1–15.2 ppm, while in unmodified **1** as well as 4-halo derivatives (**3**, **5**, **7**) shifts of the signal for the C-20 carbon atom were observed in the range 56.1–56.5 ppm.

### 3.2. In Vitro Determination of Drug-Induced Inhibition of Human Cancer Cell Line Growth

The synthesized colchicine derivatives (**2**–**8**) and starting material (**1**) were evaluated for their in vitro antiproliferative effect on normal and cancer cells. Each compound was tested on four human cancer cell lines, including one cell line displaying various level of drug resistance, namely human lung adenocarcinoma (A549), human breast adenocarcinoma (MCF-7), human colon adenocarcinoma cell line (LoVo) and doxorubicin-resistant subline (LoVo/DX). The antiproliferative effect was also studied on normal murine embryonic fibroblast cell line (BALB/3T3) for better description of cytotoxic activity of the compounds studied. The mean values of IC_50_ ± SD of the tested compounds are collected in Table 1. To evaluate the agents’ activity against the cells with MDR (multidrug resistance) phenotype, one drug resistant cancer cell line, i.e., LoVo/DX, was tested and the indexes of resistance (RI) were calculated (see Table 1). The RI values indicate how many times more resistant is the subline in comparison to its parental cell line.

All obtained derivatives with single modification at either the C-4 or C-10 position as well as double-modified compounds showed better antiproliferative activity against all tested cancer cell lines than unmodified **1** and some common chemotherapeutics such as doxorubicin and cisplatin. The IC_50_ values of novel 4-halothiocolchines are better than for single-modified colchicines in C-4 positions and remain at a level similar to the cytotoxicity of **2** for the A549 and MCF-7 cell lines.

As many as three of the compounds tested on the LoVo cell line (**6**–**8**), including two novel double-modified derivatives (**6**,**8**), exhibited extremely high activity (IC_50_ = 0.007–0.014 μM), which is even better than the activity of **2** (IC_50_ = 0.021 μM). During the tests on the doxorubicin-resistant subline (LoVo/DX), compounds **4** and **6** showed the best activity among all tested compounds. However, the *RI* values of the tested compounds indicated that colchicines did not break the drug resistance of LoVo/DX (*RI* = 9.64–278). Comparison between the cancer cell lines and the normal cell line (BALB/3T3) was made to define the Selectivity Index (*SI*) as a measure of therapeutic potential. This parameter seems to be especially important for drug-like molecules based on a scaffold of a toxic compound. The *SI* values showed that compounds **2**, **6** and **8** mostly targeted cancer cells, and fewer targeted normal cells (*SI* = 10.08–10.45, *SI* = 6.76–11.85, *SI* = 5.45–16.43 for A549, MCF-7, LoVo cancer cell lines, respectively). Also compounds **3** and **5** indicated good *SI* values for MCF-7 cell line (*SI* = 6.00, *SI* = 5.26, respectively), as well as compound **7** indicated good *SI* value for LoVo cell line (*SI* = 13.5).

### 3.3. Molecular Docking: in Silico Determination of Drug-Induced Inhibition of βI Tubulin

To further investigate the ability to inhibit tubulin aggregation by the new colchicine derivatives in cancer cell growth assays, binding energies between the new compounds and βI tubulin, one of the subunits of microtubules in the cytoskeleton structure of every eukaryotic cell, were calculated using docking methodology. The eight structures of colchicine and its derivatives described above were docked into the βI tubulin CBS and ranked according to their binding affinity (Table 2).

Since the binding energy shows how strong the interaction between the distributed drug in the cell and β-tubulin protein can be, the partition coefficient (MlogP) values were calculated and considered for the ability of the drugs to diffuse into the cells (Table 2). The MlogP values can be a beneficial factors in estimating and comparing the distribution of the novel drugs within the cells, organs and the body [40].

Based on our computational predictions, compounds **6**, **4**, **5** and **3** show the lowest binding energies of −8.6, −8.6, −8.4 and −8.3 kcal/mol, respectively. In the experimental part of the study, the lowest IC_50_ values showed **4**, **6**, **8** compounds having higher activity than **3**, **5**, **7** (Table 1). To investigate in more detail why the computational results did not show better binding energies of compound **8** than **3** and **5**, the interactions between double-modified colchicine derivatives and the CBS of βI tubulin were studied.

As shown in the diagrams representing schematic interactions of the **4** and **6** compounds with the CBS of βI tubulin Lys 352, Met 259 and Asn 258 residues interact with oxygen of the carbonyl of the C ring (sidechain acceptor), with hydrogen of thiomethyl group on ring C and hydrogen of C-11 on ring C (sidechain donor) and with ring C (arene-H), respectively.

However, Lys 352, Met 259 and Val 315 residues of the CBS of βI tubulin interact with oxygen of the carbonyl of the C ring (sidechain acceptor), with hydrogen of C-11 on ring C (sidechain donor) and with hydrogens of methoxyl groups on ring C (backbone donors) of the **3** and **5** compounds, respectively.

The study was continued for compound **8** with the highest binding energy for better understanding over the effect of interaction of βI tubulin’s residues with novel derivatives on better activities of the derivatives. Compound **8** interacts with Ala317 residue of βI tubulin, iodine on ring A (backbone donor) and Cys241 residue of βI tubulin, hydrogen of methoxyl group on ring A (sidechain donor) and Ala 316 residue of βI tubulin, the oxygen of carbonyl on ring B (sidechain acceptor).

Since the IC_50_ is a cell-based assay and the βI tubulin isotype is not the only isotype of tubulin expressed in the cell, we decided to check interactions of the synthesized compounds with the other tubulin isotypes present in the referred cell lines. Tubulin isotypes are highly conserved in all mammals as discussed by Luduena [41]. As is commonly the case, both normal and cancer cells in humans contain the same tubulin isotypes. However, their expression levels differ and specifically βIII (TUBB3) tubulin is very narrowly distributed in normal cells while it is almost always found in cancer cells and it is often correlated with drug resistance [42,43,44]. Furthermore, βI isotype (TUBB) is the most abundant isotype in most tumors, followed by, βIVb (TUBB4B), βIIa (TUBB2A), βV (TUBB6), and βIII (TUBB3) with 47%, 38%, 8.9%, 3.1%, and 2.2% respectively and with βIVa (TUBB4), βIIb (TUBB2B) and βVI (TUBB1) levels below 0.5% of the total β tubulin [42]. Interestingly, the binding sites for common tubulin-binding agents do not vary significantly between tubulin isotypes except for the CBS [8].

Tubulin isotype classes βIVa and βIVb comprised more than 50% of the total β tubulin in breast cancer (MCF-7) and colon cancer cells. The expression of βI, βIII and βIV (a and b) in the MCF-7 cell line has been reported as 39.1%, 2.5% and 58.4% respectively [45]. In another study, the ratio for MCF-7 cell line was reported as 55% to 6% to 39% [46]. In colon cancer cells, the percentage expression of the referred tubulin isotypes is 61.8%, 0.2% and 38%, respectively. The ratio of the isotype expression in lung cancer cells (A549) is given as 71.9% to 1.6% to 26.5% [45]. Consequently, each of the cell lines investigated has a distinct distribution of tubulin isotypes, which affects the overall response to a cytotoxic agent whose affinity for each of these isotypes is different making the problem of computational prediction complex.

To quantify the assumption that these compounds have different binding free energies for each of the tubulin isotypes, docking simulations between the novel colchicine derivatives and βI (UniProt ID: P07437), βIIa (UniProt ID: Q13885), βIIb (UniProt ID: Q9BVA1), βIII (UniProt ID: Q13509), βIVa (UniProt ID: P04350), βIVb (UniProt ID: P68371), βV (UniProt ID: Q9BUF5), and βVI (UniProt ID: Q9H4B7), isotypes were performed. Tubulin structure 1SA0.pdb was used as the homology model template for all tubulin isotypes using MOE2015. To visualize the results, a heat map was prepared to better illustrate the comparison of binding energies between the investigated compounds and the different tubulin isotypes using AutoDock Tableau Desktop (version 2018.2.0, Tableau Research, Standford University, Seattle, WA, USA) (see Table 3).

As can be seen in the heat map above, the binding energy between compound **8** and tubulin isotype βIIa as well as βVI are good examples of high binding energies while for compounds **3** and **5** their interaction with βI tubulin and βIIa dominates. For compound **7** βVI, βIIa and βIII are the strongest binding tubulin isotypes. These differences might be the reasons of a discrepancy between experimental and computational data. However, even data concerning the level of tubulin isotypes expression reported for the same cell line in the literature, differ from each other. Precise levels of isotype expression are not only hard to determine but they fluctuate in the same cell line as a result of exposure to various drugs [47]. Therefore, more detailed experimental studies on the isotype expression among different cell lines are still needed to give a better insight in this issue in the future.

In terms of a mechanistic explanation, specific residues of βIIa tubulin that are involved in interactions with compound **8** are Asn 256 that interacts with C-20 on ring C (backbone donor) and with ring C (arene-H) and Cys 239 that interacts with hydrogen of methoxyl group on ring A (sidechain donor). The residues of βVI tubulin that are involved in interactions with compound **8** are Asn256, which interacts with carbon atoms of two methoxyl groups on ring A (sidechain donors), Ala248, interacts with the oxygen of carbonyl on ring C (sidechain acceptor) and Asn247 that interacts with C-20 on ring C (sidechain donor) (see Table 4).

The analysis of interactions between compounds **4**, **6** and βI and compound **8** and βIIa and βVI shows that an arene-H interaction between ring C Asn256 or Asn258 and a sidechain acceptor interaction between the oxygen of carbonyl on ring C and either Lys352 or Ala248 can result in a strong binding effect. As mentioned before, the probability of the expression of βIIa in most tumors is approximately 9% versus less than 0.5% for each isotype in the group of βIVa, βVI and βIIb [42].

To the best of the authors’ knowledge of the literature, ligands binding to alpha and β tubulin are exclusive, except of course of ATP, and therefore, we do not expect any cross-interactions of our compounds with alpha tubulin [48]. Concerning interactions with ABC transporters, it is quite possible that our compounds are substrates for these multidrug resistance enzymes, but this is common to many chemotherapy drugs, including taxanes. Therefore, in order to inhibit this interaction, it would most advantageous to use our compounds in combination with some of their modulators, e.g., verapamil [49].

## 4. Discussion

We synthesized three novel double-modified 4-halothiocolchicines (**4**, **6**, **8**) and evaluated their biological activity according to the in vitro antiproliferative tests as well as the molecular docking. For a better comparison, also the activity of single-modified colchicine derivatives (**2**, **3**, **5**, **7**) as well as colchicine itself (**1**) was evaluated on four human cancer cell lines and normal murine embryonic fibroblast cell line. The results of our study clearly showed that the antiproliferative activity of novel 4-halothiocolchines (**4**, **6**, **8**) is better than the activity of 4-halocolchicines (**3**, **5**, **7**) and remain at a level similar to the cytotoxicity of **2** for the A549, MCF-7 and LoVo cell lines. Furthermore, the cytotoxicity of compounds **4**, **6** and **8** is higher than cytotoxicity of unmodified colchicine (**1**) and commonly used chemotherapeutics such as doxorubicin and cisplatin.

The introduction of thiomethyl group in C-10 position significantly increased the cytotoxicity in comparison to single-modified 4-halo derivatives (**3**, **5**, **7**) as well as allowed to reduce the toxicity for 4-bromo and 4-iodo derivatives. Compounds 4-bromothiocolchine (**6**) and 4-iodothiocolchicine (**8**) proved to be less toxic to normal murine fibroblast cells than the currently used anticancer drugs, such as cisplatin and doxorubicin, which is confirmed by their high SI values. The appropriate modification of colchicine molecule and synthesis of its analogs might overcome the toxicity, which is a major challenge in designing a potential colchicine-based drug candidate. However, it is still challenging to draw clear conclusions from the molecular-level calculations. Compounds **6**, **4**, **5** and **3** showed the lowest binding energies of −8.6, −8.6, −8.4 and −8.3 kcal/mol, respectively. The results only partially correlate with in vitro determined IC_50_ values. This may be explained by several additional effects taking place in living cells compared to the computational simulations that focus only on the binding mode of the compounds to the target. Specifically, off-target interactions involving efflux pumps with different affinities for the individual compounds may explain the observed partial correlation between IC_50_ values and binding free energies. Additionally, differences in the solubility values and membrane permeability may have to be accounted for when ranking the various compounds in biological assays and comparing them to computational predictions based on binding affinity for the target alone. We have partially addressed this issue by performing docking simulations for the remaining tubulin isotypes, several of them may be expressed in cancer cells in a manner different than in normal cells. We have demonstrated that a higher affinity for βVI tubulin of the compounds investigated may explain the differences in their biological activities. Our studies clearly show the potential of the obtained double-modified compounds. In particular, 4-halothiocolchicines are worthwhile for a continuation of the search for strong and broad-spectrum anticancer agents. Inspired by these preliminary results we plan subsequent modifications in C-7 position to obtain a series of triple-modified derivatives. Further evaluation should help to find more detailed structure-activity relationships of microtubule-targeting drugs and CBS inhibitors, which can help in rational drug design in the future.

## Figures and Tables

**Table 1:** Antiproliferative activity (IC_50_) of colchicine (**1**) and its derivatives (**2**–**8**) compared with antiproliferative activity of standard anticancer drugs doxorubicin and cisplatin and the calculated values of the resistance index *(RI)* and selectivity index (*SI*) of tested compounds [19,39].

Compound	A549		MCF-7		LoVo		LoVo/DX			BALB/3T3
	IC_50_ (μM)	*SI*	IC_50_ (μM)	*SI*	IC_50_ (μM)	*SI*	IC_50_ (μM)	*SI*	*RI*	IC_50_ (μM)
**1**	0.149 ± 0.009	1.4	0.128 ± 0.135	1.6	0.108 ± 0.025	1.9	2.65 ± 0.96	0.1	24.5	0.208 ± 0.042
**2**	0.011 ± 0.001	10.1	0.010 ± 0.002	11.9	0.021 ± 0.006	5.5	0.398 ± 0.075	0.3	19.0	0.114 ± 0.072
**3**	0.046 ± 0.035	3.0	0.023 ± 0.005	6.0	0.069 ± 0.012	2.0	0.784 ± 0.28	0.2	11.4	0.138 ± 0.069
**4**	0.022 ± 0.002	1.0	0.022 ± 0.002	1.0	0.022 ± 0.002	1.0	0.111 ± 0.044	0.2	5.1	0.022 ± 0.002
**5**	0.105 ± 0.008	1.4	0.027 ± 0.008	5.3	0.084 ± 0.021	1.7	1.55 ± 0.17	0.1	18.5	0.142 ± 0.073
**6**	0.010 ± 0.0001	10.3	0.015 ± 0.002	6.9	0.014 ± 0.004	7.4	0.135 ± 0.012	0.8	9.6	0.103 ± 0.089
**7**	0.094 ± 0.006	1.4	0.098 ± 0.029	1.4	0.010 ± 0.002	13.5	2.78 ± 0.45	0.1	278.0	0.135 ± 0.056
**8**	0.011 ± 0.002	10.5	0.017 ± 0.006	6.8	0.007 ± 0.002	16.4	0.642 ± 0.084	0.2	91.7	0.115 ± 0.044
Doxorubicin	0.258 ± 0.044	0.6	0.386 ± 0.118	0.4	0.092 ± 0.018	1.8	4.75 ± 0.99	<0.1	51.6	0.166 ± 0.074
Cisplatin	6.367 ± 1.413	0.6	10.70 ± 0.753	0.4	4.37 ± 0.73	0.9	5.70 ± 0.63	0.7	1.3	3.90 ± 1.50

The IC_50_ value is defined as the concentration of a compound at which 50% growth inhibition is observed. Human lung adenocarcinoma (A549), human breast adenocarcinoma (MCF-7), human colon adenocarcinoma cell line (LoVo) and doxorubicin-resistant subline (LoVo/DX), normal murine embryonic fibroblast cell line (BALB/3T3). The *SI* (Selectivity Index) was calculated for each compound using the formula: *SI* = IC_50_ for normal cell line BALB/3T3/IC_50_ for respective cancerous cell line. A beneficial *SI* > 1.0 indicates a drug with efficacy against tumor cells greater than the toxicity against normal cells. The *RI* (Resistance Index) indicates how many times a resistant subline is chemoresistant relative to its parental cell line. The *RI* was calculated for each compound using the formula: *RI* = IC_50_ for LoVo/DX/IC_50_ for LoVo cell line. When *RI* is 0–2, the cells are sensitive to the compound tested, *RI* in the range 2–10 means that the cell shows moderate sensitivity to the drug tested, *RI* above 10 indicates strong drug resistance.

**Table 2:** Binding energies interactions between eight different colchicine derivatives and βI tubulin and the estimated Moriguchi octanol-water partition coefficient, MlogP for each colchicine derivative.

Structure	Interactions	Binding Energy (Kcal/mol)	MlogP	Active Residues
**1** 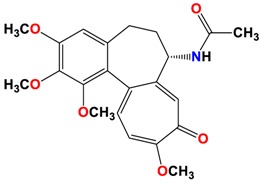	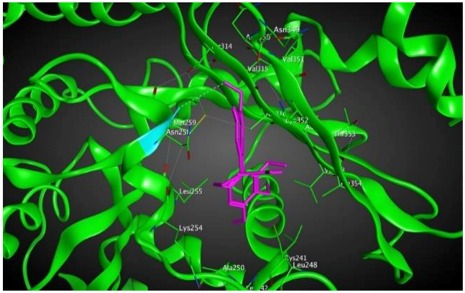	−8.09	1.37	Asn258
**2** 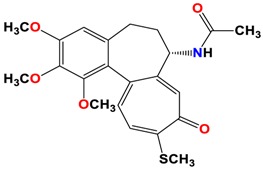	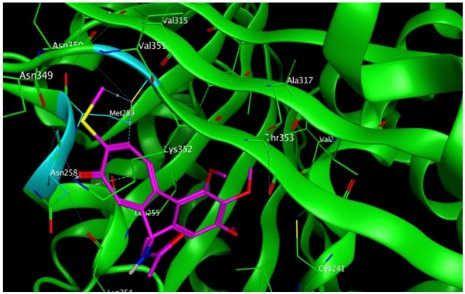	−8.13	1.56	Met259 Asn258 Lys352
**3** 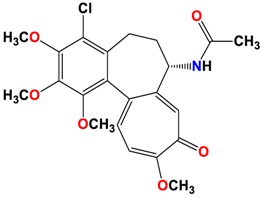	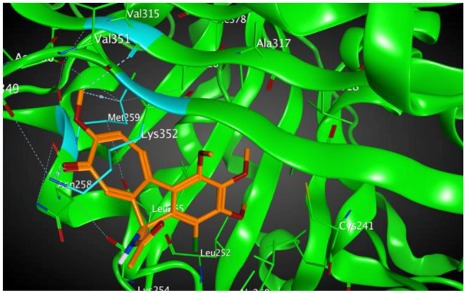	−8.33	1.58	Met259 Lys352 Val315
**4** 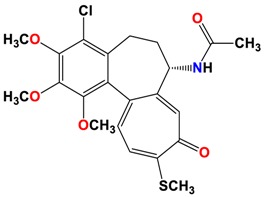	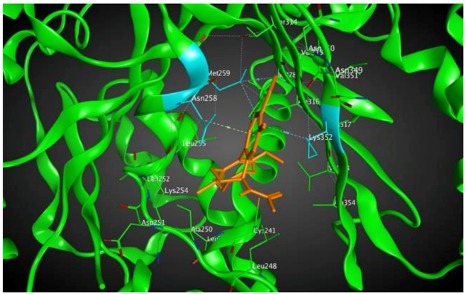	−8.57	2.34	Met259 Asn258 Lys352
**5** 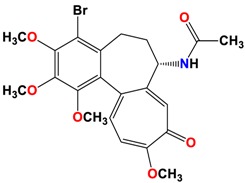	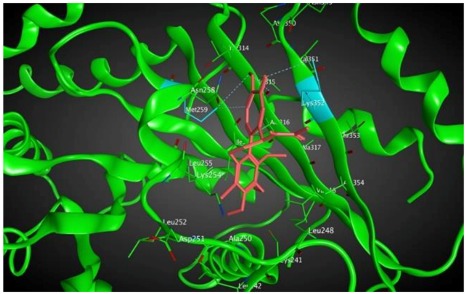	−8.40	1.93	Met259 Val315 Lys352
**6** 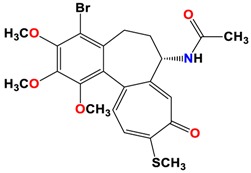	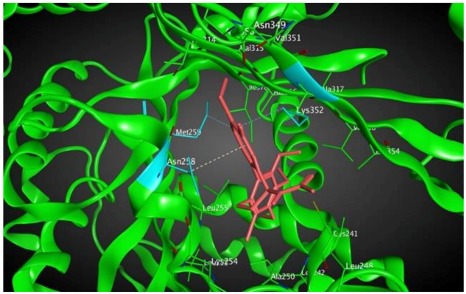	−8.60	2.69	Met259 Asn258 Lys352
**7** 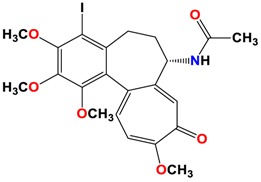	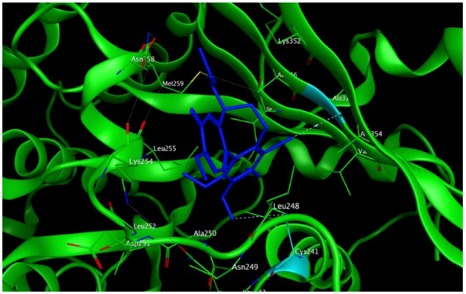	−7.53	1.50	Ala317 Cys241
**8** 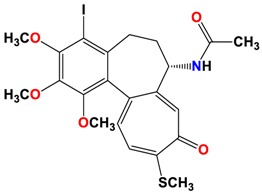	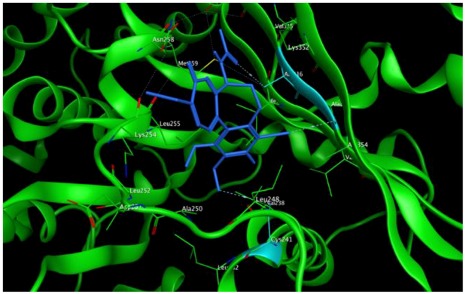	−7.33	2.00	Ala316 Ala317 Cys241

**Table 3:** Heat map of binding energies interactions between the eight different colchicine derivatives and βI, βIII βIIa, βIIb, βIVa, βIVb, βV and βVI tubulin isotypes.

	1	2	3	4	5	6	7	8
βI	−8.090	−8.130	−8.330	−8.570	−8.400	−8.600	−7.530	−7.330
βIIa	−7.420	−7.190	−7.890	−7.640	−8.000	−7.460	−8.300	−8.200
βIIb	−7.050	−6.680	−6.800	−6.900	−6.890	−7.050	−6.430	−7.040
βIII	−7.490	−7.150	−7.470	−7.620	−7.850	−7.780	−8.200	−7.710
βIVa	−7.610	−7.300	−7.100	−7.300	−7.210	−7.430	−6.970	−7.010
βIVb	−7.260	−7.210	−7.420	−7.610	−7.370	−7.620	−7.180	−6.340
βV	−7.480	−7.180	−7.320	−7.260	−7.250	−7.340	−6.500	−7.190
**βVI**	−7.730	−7.050	−7.270	−7.710	−7.590	−7.870	−8.320	−8.300

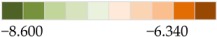								

**Table 4:** Binding energies interactions between compound **8** and βIIa and βVI tubulin as well as the estimated Moriguchi octanol-water partition coefficient, MlogP for compound **8**.

Tubulin Isotype	Interactions	Binding Energy (Kcal/mol)	MlogP	Active Residues
βIIa	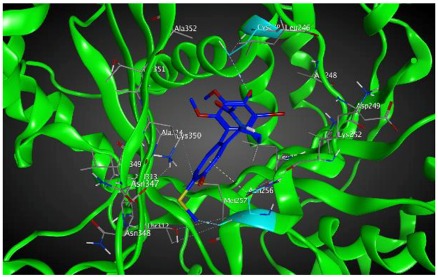	−8.20	1.99	Asn256 Cys239
βVI	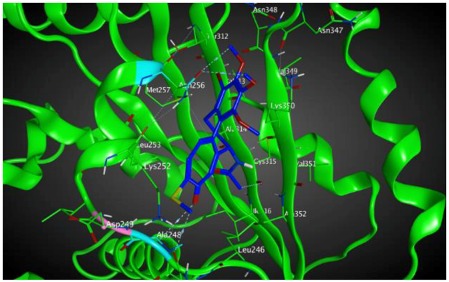	−8.30	1.99	Asn256 Asn247 Ala248

## Supplementary Materials

The following are available online, Figure S1: The ^13^C-NMR spectrum of **2** in CDCl_3_, Figure S2: The ^1^H-NMR spectrum of **2** in CDCl_3_, Figure S3: The ^13^C-NMR spectrum of **3** in CDCl_3_, Figure S4: The ^1^H-NMR spectrum of **3** in CDCl_3_, Figure S5: The ^13^C-NMR spectrum of **4** in CDCl_3_, Figure S6: The ^1^H-NMR spectrum of **4** in CDCl_3_, Figure S7: The ^13^C-NMR spectrum of **5** in CDCl_3_, Figure S8: The ^1^H-NMR spectrum of **5** in CDCl_3_, Figure S9: The ^13^C-NMR spectrum of **6** in CDCl_3_, Figure S10: The ^1^H-NMR spectrum of **6** in CDCl_3_, Figure S11: The ^13^C-NMR spectrum of **7** in CDCl_3_, Figure S12: The ^1^H-NMR spectrum of **7** in CDCl_3_, Figure S13: The ^13^C-NMR spectrum of **8** in CDCl_3_, Figure S14: The ^1^H-NMR spectrum of **8** in CDCl_3_.
