# Supplementary material for: Antiproliferative Activity and Molecular Docking of Novel Double-Modified Colchicine Derivatives
Source: Cells. 2018 Nov 1;7(11):192. doi: 10.3390/cells7110192 (PMC6262536; doi:10.3390/cells7110192)
Supplement: Supplementary file 1 [file cells-07-00192-s001.pdf]

cells

Supplementary material

Antiproliferative activity and molecular docking of novel double-modified colchicine derivatives

Urszula Majcher [1], Greta Klejborowska [1], Mahshad Moshari [2], Ewa Maj [3], Joanna Wietrzyk [3], Franz Bartl [4], Jack A. Tuszynski [2], and Adam Huczyński [1,*]

[1] Department of Bioorganic Chemistry, Faculty of Chemistry, Adam Mickiewicz University, Umultowska 89b, 61-614 Poznan, Poland;
[2] Depertment of Oncology, University of Alberta, Edmonton, Alberta T6G 1Z2, Canada;
[3] Hirszfeld Institute of Immunology and Experimental Therapy, Polish Academy of Sciences, Rudolfa Weigla 12, 53-114 Wrocław, Poland;
[4] Institut für Biologie, AG Biophysikalische Chemie,– Humboldt Universität zu Berlin, Invalidenstr, 42, 10099 Berlin, Germany;
* Correspondence: adhucz@amu.edu.pl; Tel.: +48-61-829-1673

Supplementary material

Figure S1. The ^{13}C NMR spectrum of **2** in $CDCl_3$.
Figure S2. The ^{1}H NMR spectrum of **2** in $CDCl_3$.
Figure S3. The ^{13}C NMR spectrum of **3** in $CDCl_3$.
Figure S4. The ^{1}H NMR spectrum of **3** in $CDCl_3$.
Figure S5. The ^{13}C NMR spectrum of **4** in $CDCl_3$.
Figure S6. The ^{1}H NMR spectrum of **4** in $CDCl_3$.
Figure S7. The ^{13}C NMR spectrum of **5** in $CDCl_3$.
Figure S8. The ^{1}H NMR spectrum of **5** in $CDCl_3$.
Figure S9. The ^{13}C NMR spectrum of **6** in $CDCl_3$.
Figure S10. The ^{1}H NMR spectrum of **6** in $CDCl_3$.
Figure S11. The ^{13}C NMR spectrum of **7** in $CDCl_3$.
Figure S12. The ^{1}H NMR spectrum of **7** in $CDCl_3$.
Figure S13. The ^{13}C NMR spectrum of **8** in $CDCl_3$.
Figure S14. The ^{1}H NMR spectrum of **8** in $CDCl_3$.

cells

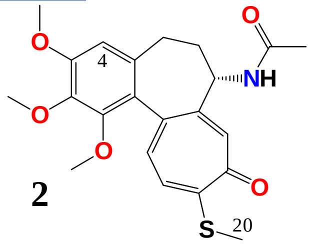

2

Chemical Formula: $C_{22}H_{25}NO_5S$
Exact Mass: 415,15

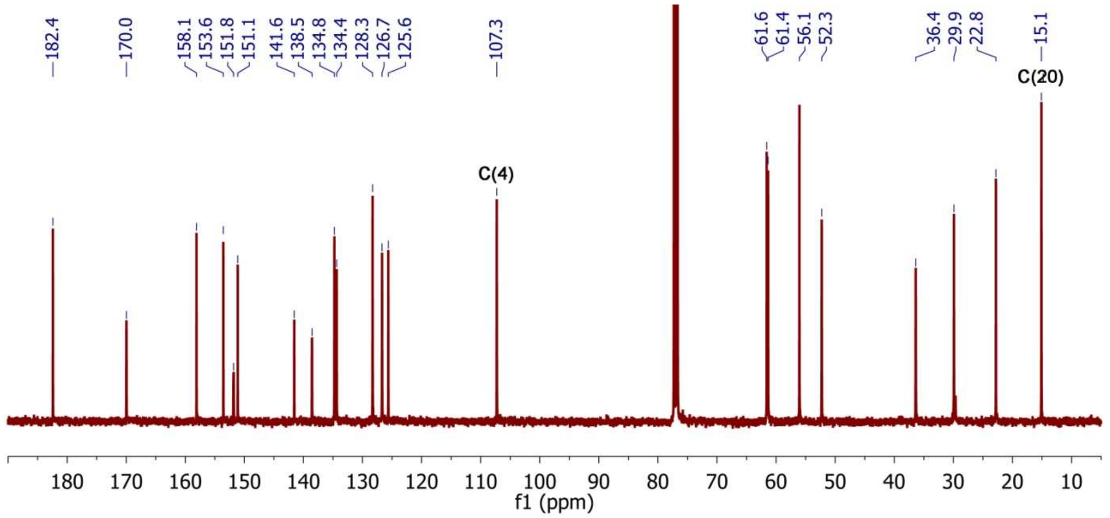

Figure S1. The ^{13}C NMR spectrum of **2** in $CDCl_3$.

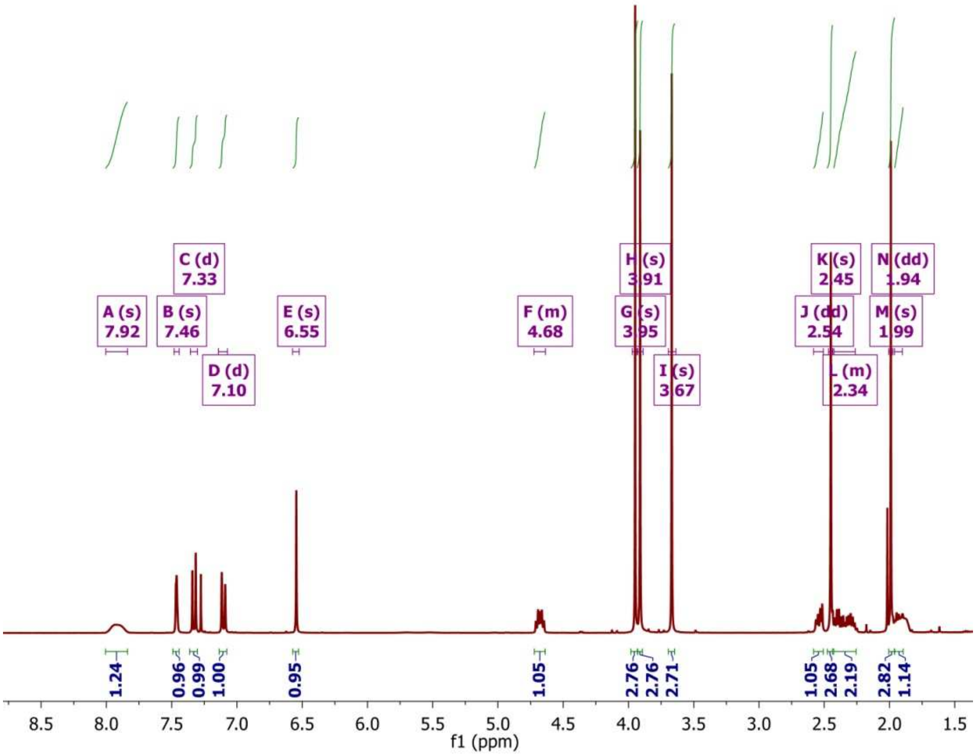

Figure S2. The ^{1}H NMR spectrum of **2** in $CDCl_3$.

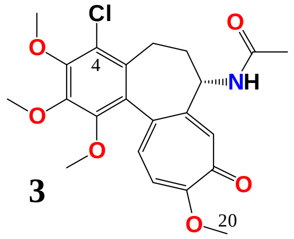

3

Chemical Formula: $C_{22}H_{24}ClNO_6$
Exact Mass: 433,13

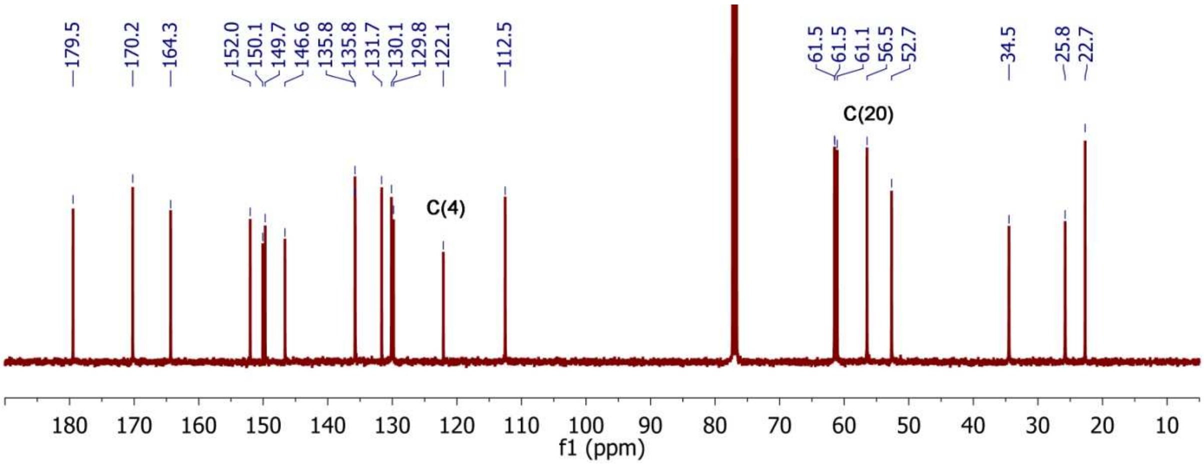

Figure S3. The ^{13}C NMR spectrum of **3** in $CDCl_3$.

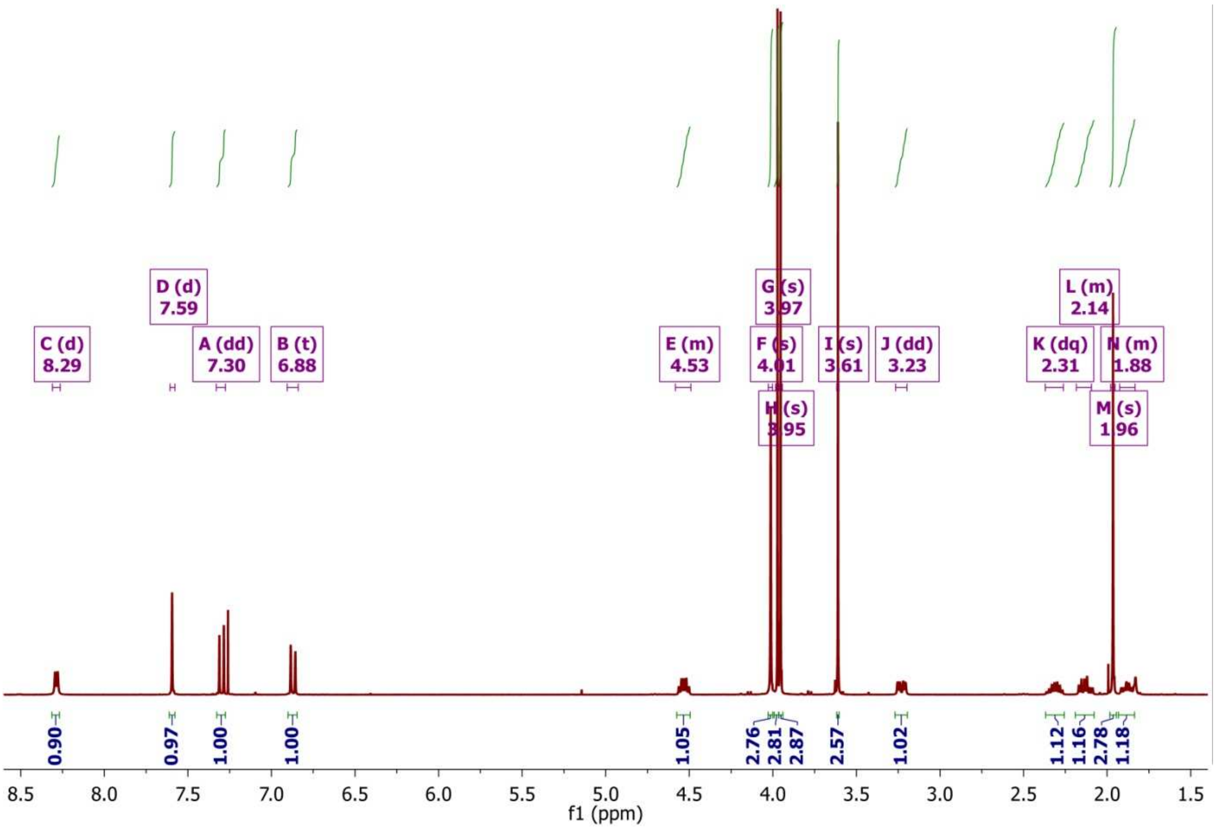

Figure S4. The ^{1}H NMR spectrum of **3** in $CDCl_3$.

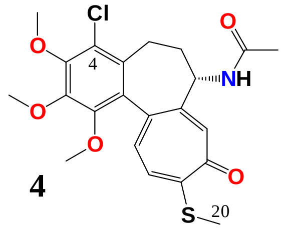

Chemical Formula: $C_{22}H_{24}ClNO_5S$
Exact Mass: 449,11

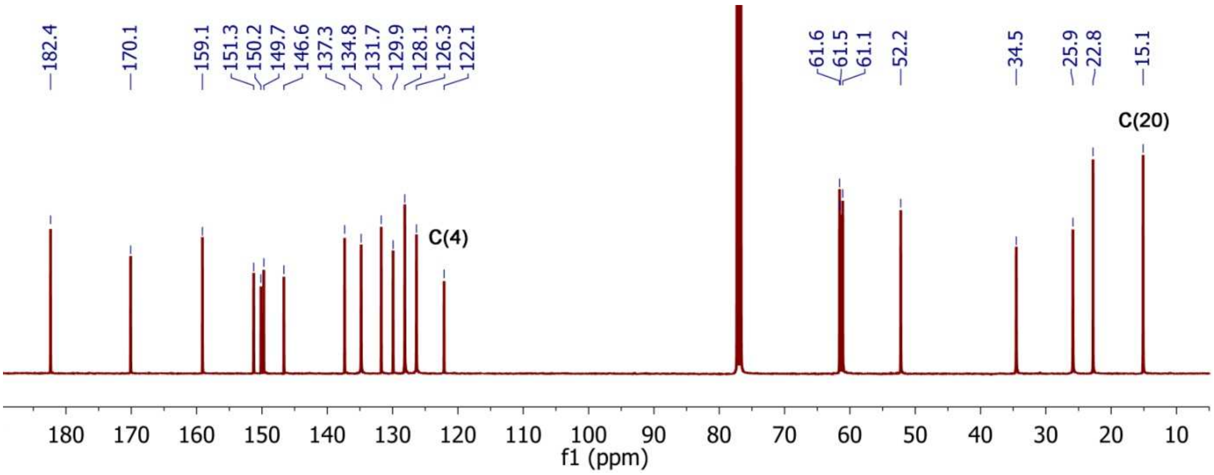

Figure S5. The ^{13}C NMR spectrum of **4** in $CDCl_3$.

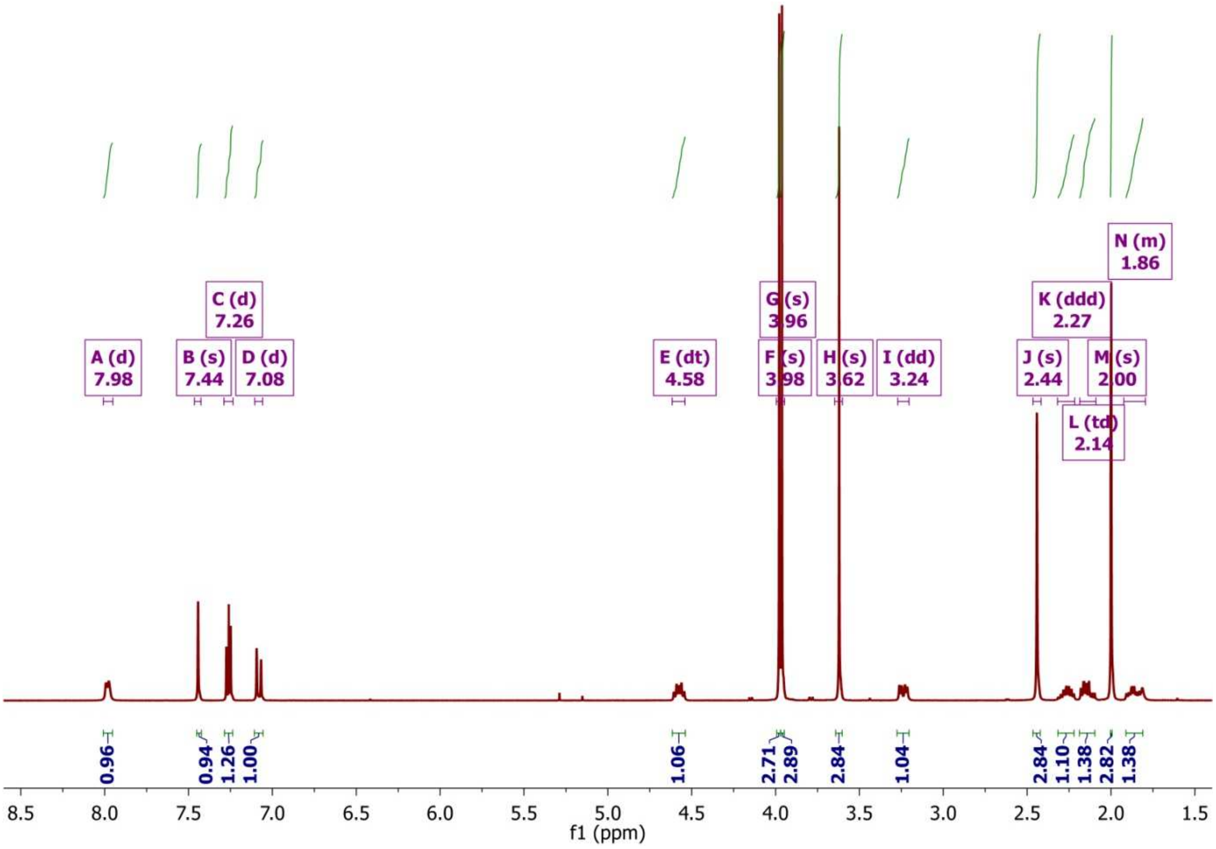

Figure S6. The ^{1}H NMR spectrum of **4** in $CDCl_3$.

cells

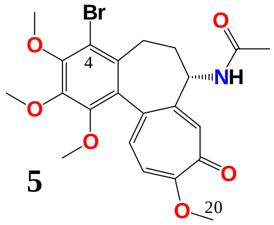

5

Chemical Formula: $C_{22}H_{24}BrNO_6$
Exact Mass: 477,08

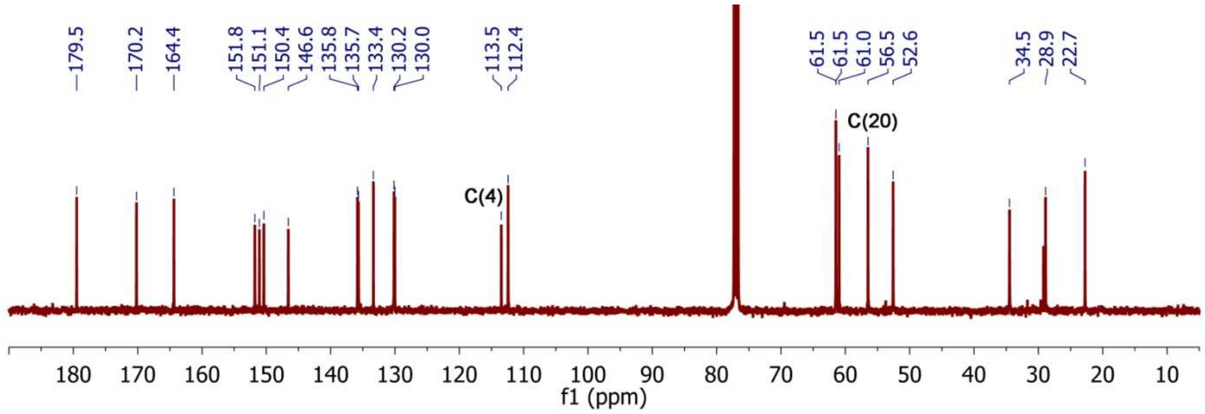

Figure S7. The ^{13}C NMR spectrum of **5** in $CDCl_3$.

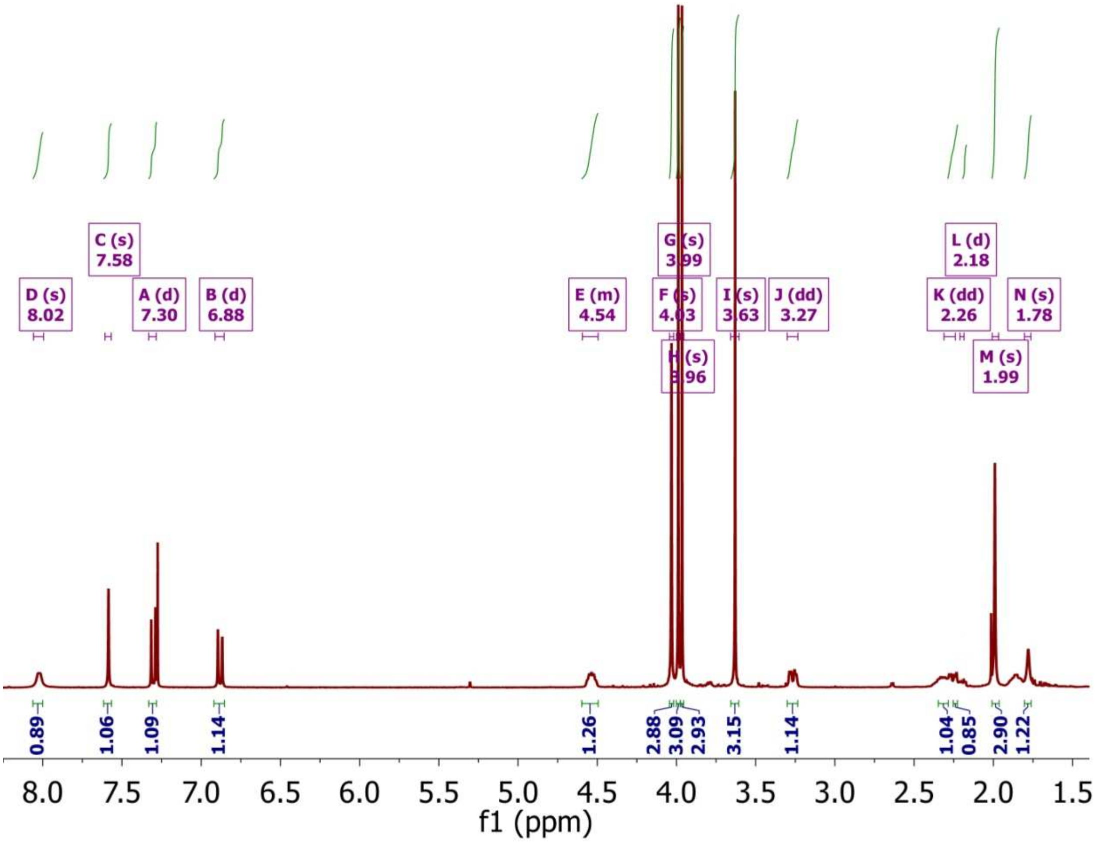

Figure S8. The ^{1}H NMR spectrum of **5** in $CDCl_3$.

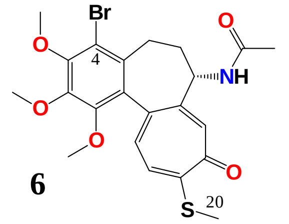

6

Chemical Formula: $C_{22}H_{24}BrNO_5S$
Exact Mass: 493,06

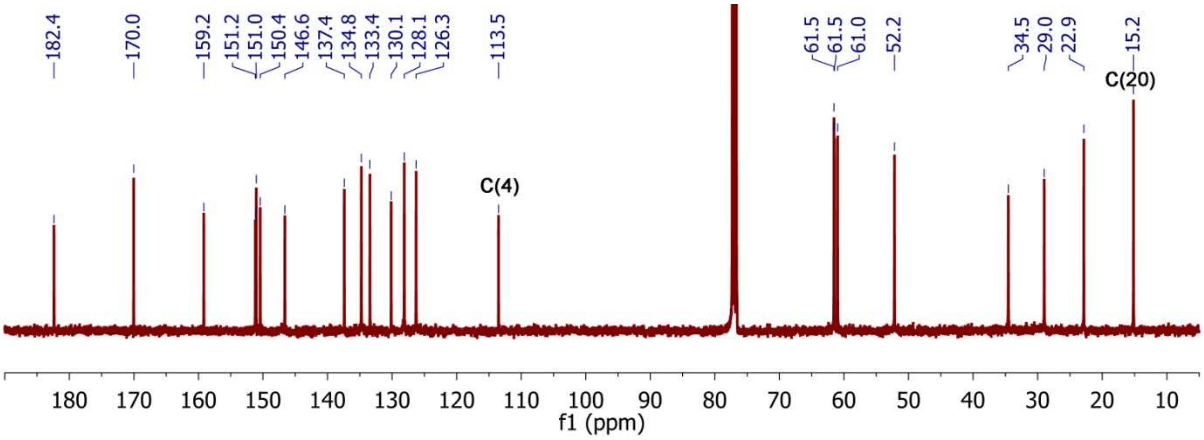

Figure S9. The ^{13}C NMR spectrum of **6** in $CDCl_3$.

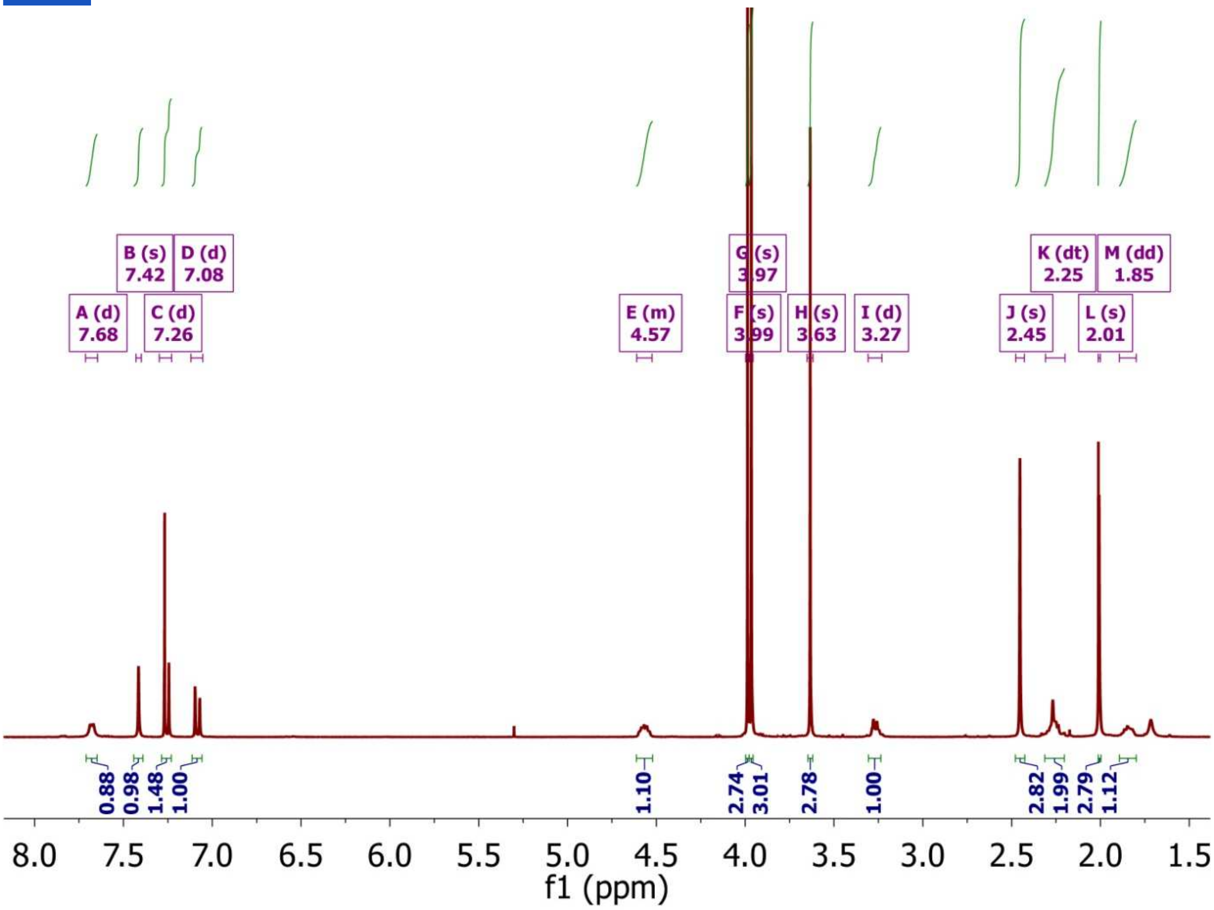

Figure S10. The ^{1}H NMR spectrum of **6** in $CDCl_3$.

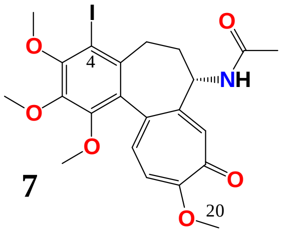

7

Chemical Formula: $C_{22}H_{24}INO_6$

Exact Mass: 525,06

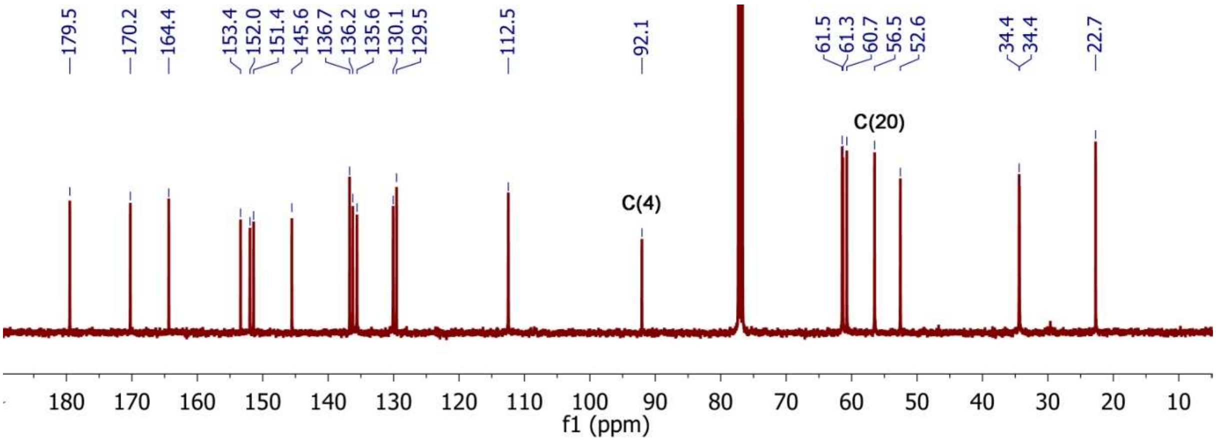

Figure S11. The ^{13}C NMR spectrum of **7** in $CDCl_3$.

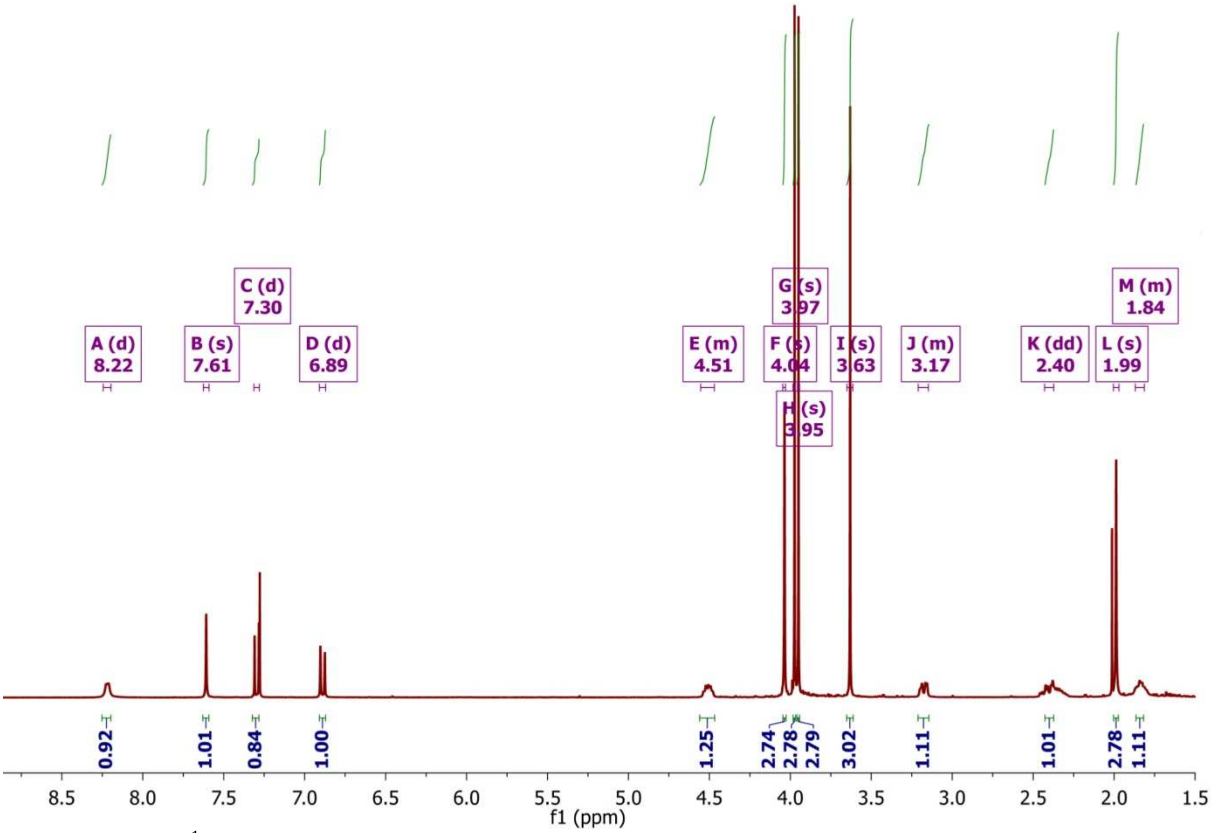

Figure S12. The ^{1}H NMR spectrum of **7** in $CDCl_3$.

cells

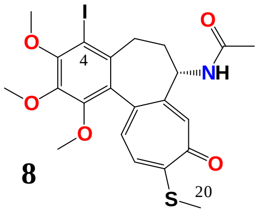

8

Chemical Formula: $C_{22}H_{24}INO_5S$
Exact Mass: 541,04

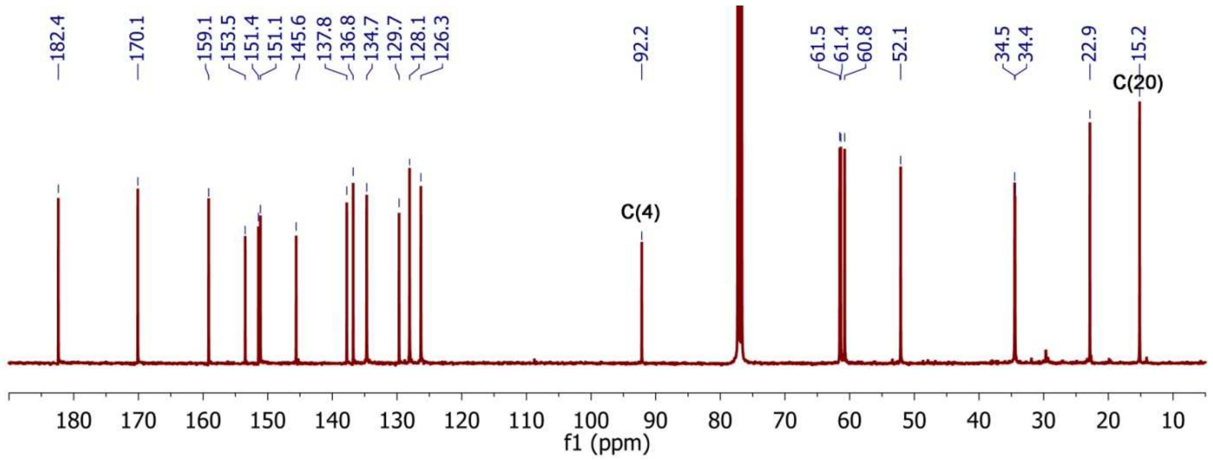

Figure S13. The ^{13}C NMR spectrum of **8** in $CDCl_3$.

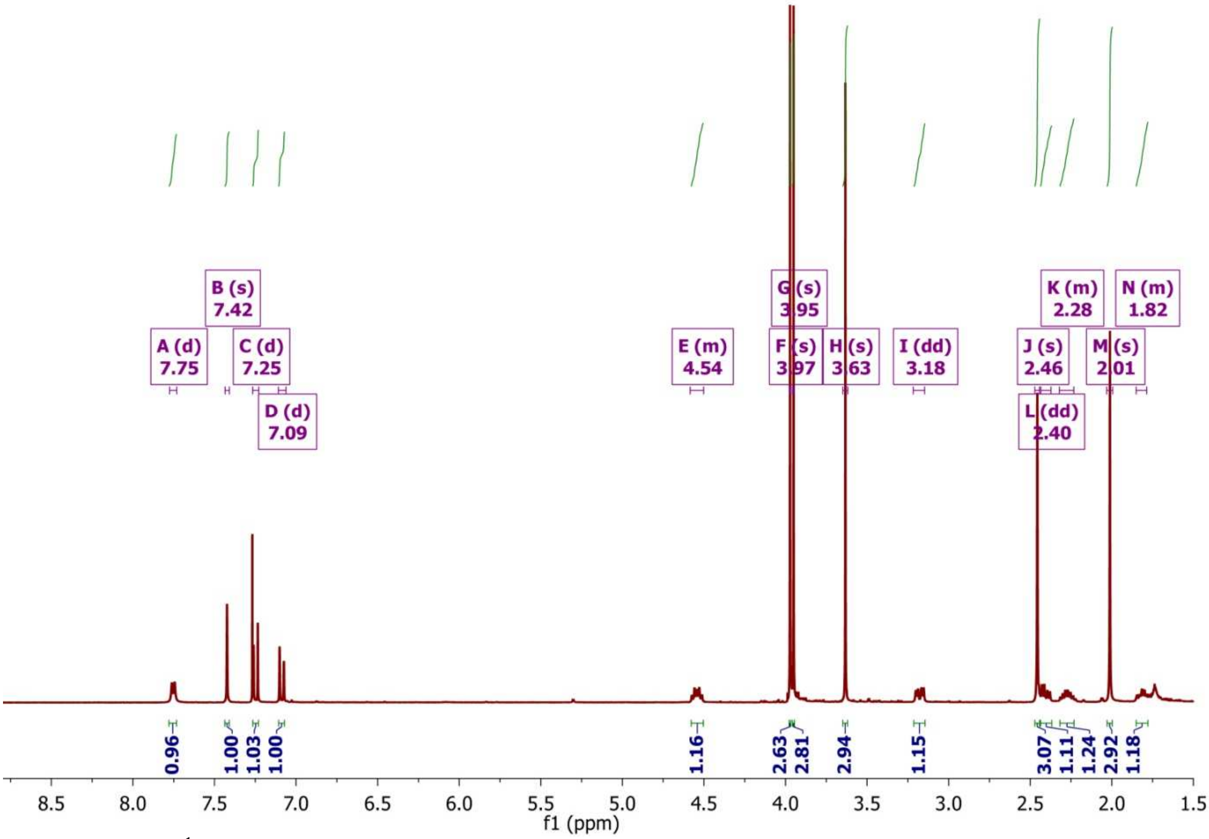

Figure S14. The ^{1}H NMR spectrum of **8** in $CDCl_3$.
